# Congenital hyperreninemic hypoaldosteronism due to aldosterone synthase deficiency type I in a Portuguese patient – Case report and review of literature

**DOI:** 10.20945/2359-3997000000107

**Published:** 2019-02-01

**Authors:** Adriana de Sousa Lages, Beatriz Vale, Patrícia Oliveira, Rita Cardoso, Isabel Dinis, Francisco Carrilho, Alice Mirante

**Affiliations:** 1 University Center Coimbra Hospital Diabetes and Metabolism Department Coimbra Portugal Endocrinology, Diabetes and Metabolism Department, Coimbra Hospital and University Center, Coimbra, Portugal; 2 University Center Coimbra Hospital Pediatric Unit Coimbra Portugal Pediatric Unit, Coimbra Hospital and University Center, Coimbra, Portugal; 3 University Center Coimbra Hospital Pediatric Endocrinology Unit Coimbra Portugal Pediatric Endocrinology Unit, Coimbra Hospital and University Center, Coimbra, Portugal

## Abstract

Hyperreninemic hypoaldosteronism due to aldosterone synthase (AS) deficiency is a rare condition typically presenting as salt-wasting syndrome in the neonatal period. A one-month-old Portuguese boy born to non-consanguineous parents was examined for feeding difficulties and poor weight gain. A laboratory workup revealed severe hyponatremia, hyperkaliaemia and high plasma renin with unappropriated normal plasma aldosterone levels, raising the suspicion of AS deficiency. Genetic analysis showed double homozygous of two different mutations in the *CYP11B2* gene: p.Glu198Asp in exon 3 and p.Val386Ala in exon 7. The patient maintains regular follow-up visits in endocrinology clinics and has demonstrated a favourable clinical and laboratory response to mineralocorticoid therapy. To our knowledge, this is the first Portuguese case of AS deficiency reported with confirmed genetic analysis.

## INTRODUCTION

Aldosterone is a mineralocorticoid hormone secreted exclusively in the zona glomerulosa (ZG) of the adrenal cortex and acts in distal renal tubules by increasing sodium reabsorption and promoting urinary potassium excretion. The aldosterone synthesis is dependent on aldosterone synthase (AS), an enzyme encoded by the *CYP11B2* gene, one of the cytochrome P450 enzymes (P450c11Aldo). AS, previously named corticosterone methyloxidase is expressed in the adrenal cortex and is responsible for catalysing the final three steps of the aldosterone biosynthesis: from 11-desoxycorticosterone (DOC) to corticosterone, then to 18-hidroxicorticosterone (18-OHB) and finally to aldosterone (1,2).

Congenital hyperreninemic hypoaldosteronism due to defects in AS caused by inactivating mutations in the *CYP11B2* gene has an unknown prevalence and is usually associated with an autosomal recessive pattern of inheritance. Two distinct forms of AS deficiency have been described, type I and type II, based only on the biochemical profile: no AS catalytic activity and low 18-hydroxycorticosterone (18-OHB) levels in AS deficiency type I and residual enzymatic catalytic activity and elevated 18-OHB levels in AS deficiency type II (3).

Typically, the disease manifests in the first weeks of life with nausea, vomiting, feeding problems and failure to thrive in the neonatal period. Isolated aldosterone deficiency is associated with neonatal salt-wasting syndrome resulting in hyponatremia, hyperkalaemia, metabolic acidosis and marked elevated renin with low or unappropriated normal aldosterone levels (4–15). Usually, clinical severity improves with age and patients are frequently asymptomatic during adulthood despite not having mineralocorticoid therapy (16).

Most of the cases reported in the literature are from Iranian Jewish patients (5,16,17), but cases from Turkey (14,18), Japan (7,8) and Israel (9,19) have also been described. To our knowledge, there has been no previous report of Portuguese cases of AS deficiency, especially with confirmed genetic analysis.

## CASE PRESENTATION

We report a case of a one-month-old infant delivered by caesarean section at 35 weeks from a dichorionic diamniotic twin pregnancy with a birth weight of 2,350g and a length of 46 cm. The parents are Portuguese and non-consanguineous.

Our patient presented 1-week complaints of an increased number of dejections and vomiting predominantly after feeding. At the first examination in a local paediatric hospital, he presented severe signs of dehydration (heart rate 160 bpm, blood pressure 92/58 mmHg, abnormally prolonged capillary refill time, deeply sunken eyes, mottled extremities), failure to retrieve weight compared to birth weight (2,340g) and marked hypotonia. He had a normal male phenotype with no genital anomalies, and no skin hyperpigmentation was described. A laboratory examination revealed an elevated plasma potassium level of 7.4 mmol/L [normal range (NR) 3.5-5.1], a low plasma sodium level of 126 mmol/L (NR 137-145) and metabolic acidosis (pH 7.16 pCO2 46 mmHg HCO3^−^ 16.4 mmol/L Lactates 5.3 mmol/L Anion Gap −13.6 mmol/L). Prompt therapy with 5% dextrose and 0.45% sodium chloride (NaCl) as well as infusion of sodium bicarbonate were initiated, and the patient was transferred to our central medical unit.

During the hospital admission, because of a clinical presumptive diagnose of congenital adrenal hyperplasia, he was given therapy with hydrocortisone (2.5 mg 6/6h) and oral NaCl supplement (0.8 mEq/kg/day) with initial favourable clinical and laboratory response.

However, a hormonal workup revealed normal plasma levels of adrenocorticotropic hormone (ACTH) (11.8 pg/mL – NR < 46), cortisol (10.9 µg/dL – NR 5.0-25.0), 17-OH-progesterone (17-OHP) (10.0 ng/mL), androstenedione (0.9 ng/mL) and dehydroepiandrosterone sulfate (DHEA-SO4) (32.5 µg/dL) with marked elevated renin (> 500 µUI/mL) levels and unappropriated normal aldosterone [12.4 ng/dL; measured by chemiluminescent immunoassay (CLIA) on Liaison®], ruling out the first clinical suspicion of congenital adrenal hyperplasia (CAD) and raising the suspicion of primary hypoaldosteronism as the probable cause of the severe neonatal salt-wasting syndrome (Table 1). Ultrasound investigation of the kidneys revealed no abnormalities, and there were no signs of enteropathy, urinary tract infection or obstruction.

**Table 1:** Initial and sequential plasma levels (ionogram and hormonal profile) in Portuguese patient with congenital hyperreninemic hypoaldosteronism due to aldosterone synthase deficiency

	13/10/2012 presentation	5/11/2012	13/12/2013	02/01/2014	7/10/2014	21/4/2016	13/3/2018	Normal range
**Weight (g)**	2,340	2,980	4,240	4,740	10,100	12,900	17.400	
**Height (cm)**	46		56	57.5	78.5	95.1	106.4	
**Age**	31 days	54 days	91 days	110 days	18 months	3 years+7 months	5 years+6 months	
**Basal plasma levels**								
Na^+^ (mmol/L)	**126**	140	141	139	140	142	140	137-145
K^+^ (mmol/L)	**7.4**	**5.95**	**5.49**	4.61	4.06	4.39	4.1	3.5-5.1
ACTH (pg/mL)	11.8							< 46.0
Basal cortisol (µg/dL)	10.9							5.0-25.0
**Basal 17-OHP** (ng/mL)	10.0							
**Renin** (µUI/mL) (uU/mL)	**> 500**	**> 500**	**208.2**	26.5	**0.5**	**1.9**	**0.5**	1.0-16.0 7-76
**Aldosterone** (ng/dL) (pg/mL)	12.4	9.8	6.4	1.1	1.2	**16.2**	**24.6**	2.8-39.9 40-310
**Free testosterone** (pg/mL)	2.81							
**Androstenedione** (ng/mL)	0.9							
**DHEA-SO**_4_ (µg/dL)	32.5							
**Therapy**	–	Oral 20% NaCl; Exchange resin sodic phase	Oral 20% NaCl supplement; Fludrocortisone 0.05 + 0.025 mg	Oral 20% NaCl supplement; Fludrocortisone 0.05 + 0.025 mg	Oral 20% NaCl supplement; Fludrocortisone 0.05 + 0.025 mg	Fludrocortisone 0.05 + 0.025 mg	Fludrocortisone 0.05 + 0.025 mg	

ACTH: adrenocorticotropic hormone; 17-OHP: 17-OH-progesterone; DHEA-SO_4_: dehydroepiandrosterone sulfate.

The patient was discharged with an oral 20% NaCl supplement (0.4 mL, 8 times/day after feeding) and ion-exchange resin – sodic phase (1.8g, 3 times/day). At 54 days old, he had his first paediatric endocrinology appointment with new laboratory evaluation, confirming the results previously obtained and maintenance of an elevated plasma potassium level of 5.95 mmol/L (NR 3.5-5.1), so therapy with fludrocortisone (0.075 mg/day, 0.018 mg/kg/day) and NaCl supplementation were associated.

Genetic analysis was performed with direct DNA sequence analysis of the entire coding regions of the *CYP11B2* gene and revealed that our patient is a double-homozygous carrier of two mutations: in exon 3, codon 198 with replacement of glutamic acid for aspartic acid - c.594A > C (p.Glu198Asp), and in exon 7, codon 386 with replacement of valine for alanine - c.1157T > C (p.Val386Ala). Genotyping of these variations in both parents revealed that both were heterozygous carriers of the same two mutations, c.594A > C (p.Glu198Asp) and c.1157T > C (p.Val386Ala) – mutation *in cis* – which implies a 25% risk of AS deficiency in offspring.

The patient maintains regular follow-up visits to endocrinology clinics, is currently 5 years old and good clinical effect on body growth and normalization of laboratory abnormalities after introduction of mineralocorticoid therapy. The dose of oral fludrocortisone has been stable at 0.075 mg/day, in two divided doses.

## DISCUSSION

Aldosterone is secreted on the ZG of the adrenal gland under control of three principal factors: angiotensin II, potassium and, to a lesser extent, ACTH. The first two secretagogues stimulate aldosterone secretion mainly by increasing the transcription of the *CYP11B2* gene. Distinctly, ACTH acutely stimulates the early pathways of adrenal steroidogenesis, promoting a 10%-20% elevation of the aldosterone circulating levels (20).

Mineralocorticoid deficiency can be divided in two categories: congenital and acquired syndromes.

Mutations in the gene encoding the AS enzyme, exclusively found in the ZG, are associated with isolated mineralocorticoid deficiency. Nevertheless, functional mineralocorticoid deficiency may also be related to mutations in the mineralocorticoid receptor or in the gene encoding the epithelial sodium channel (ENaC) (21).

Regarding primary defects in aldosterone biosynthesis, mutations in AS limit the activation of DOC to aldosterone through three terminal subsequent steps catalysed by the mitochondrial CYP enzyme 11B2 (CYP11B2). Patients with AS deficiency can be classified as type I or type II according to the variable biochemical phenotype (based on 18-OHB and aldosterone levels). Both cases are rare and are inherited in an autosomal recessive pattern (3).

Our patient presented with severe salt-wasting syndrome with important signs and symptoms of dehydration, feeding difficulties and failure to thrive in the neonatal period. Clinical presentation along with a typical biochemical profile with hyponatremia, hyperkalaemia, normal cortisol and sex steroids, high plasma renin levels and unappropriated normal aldosterone levels suggested the underlying diagnosis, posteriorly confirmed by genetic analysis of *CYP11B2* gene mutations on the index case.

In the neonatal period, clinical presentation can be dramatic, with significant hemodynamic impact and life-threating episodes of seizure and coma. Typically, we assist an improving of clinical severity with age, and adults are frequently asymptomatic despite no mineralocorticoid therapy. This fact can be associated with a gradual declining dependency on aldosterone during lifetime for normal ionic imbalance due to possible extra-adrenal compensatory mechanisms and alternative ACTH-dependent pathways for mineralocorticoid biosynthesis (22).

Occasionally, during adulthood, patients can present with orthostatic hypotension and hyperkalaemia in particular situations (as dehydration and heat stroke). A complete past medical history, with detailed childhood development and other medical conditions associated (such as nausea, vomiting, feeding problems and deficit in weight gain), is essential to identify the correct diagnosis (1,16,23).

It is also important to establish a correct differential diagnose with another neonatal salt-wasting syndromes such as congenital adrenal hyperplasia (CAH). In the latter, besides the clinical similarity at presentation, patients frequently have genital abnormalities (clitoral hypertrophy, labia partially or completely fused in girls) and a hormonal profile that is clearly distinct – instead of an isolated aldosterone deficiency with exclusively mineralocorticoid deficiency, on most patients (> 95%) with CAD due to 21-hydroxylase deficiency (CYP21A2), we found a combined aldosterone and cortisol synthesis deficiency associated with raised 17-OHP, with elevated adrenal androgens as a hallmark of the diagnosis (24). Within this line, the therapy strategy is different and must include life-long glucocorticoid and mineralocorticoid replacement (16).

Typically, patients with AS deficiency respond well to fludrocortisone therapy and may also benefit from salt supplementation specifically in the neonatal period. In our patient, NaCl supplementation was discontinued at 2 years of age, and maintenance of mineralocorticoid therapy with fludrocortisone in a stable dose of 0.075 mg allowed a catch-up growth with a favourable clinical and biochemical response.

Generally, genetic analysis of AS deficiency cases identified mutations on the *CYP11B2* gene. Our patient is a double-homozygous carrier for two sequence changes leading to amino acid substitutions: E198D in exon 3 and V386A in exon 7. Both parents are heterozygous carriers for the same two mutations, which justifies the fact that neither have any clinical manifestation of the disease – mutation *in cis*. The combination of these two mutations encodes enzymes with a reduced residual 11β-hydroxylase activity; however, with no broad catalytic activity, the clinical phenotype arises (25).

Although most cases of AS deficiency reported a mutation on the *CYPB11B2* gene, this condition has recently been associated with cases of patients with clinically and biochemically presumed AS deficiency with no identified mutations on the *CYP11B2* gene, emphasising a bigger heterogeneity and possible other mechanisms associated with the disease – mutations in genes encoding other renin-aldosterone system components (such as angiotensinogen, angiotensin-converting enzymes and angiotensin II-receptor) or other regulatory genes of AS function. Encouraging the report of cases not linked to the *CYPB11B2* gene mutations is important precisely to estimate the proportion of patients without positive genetic analysis and contribute to a broad comprehension of the molecular basis of the condition (6,9).

Unfortunately, in our country, we did not have access to measurement of plasma or urinary 18-OHB levels and corticosterone levels that clearly distinguish AS deficiency type I from type II. However, the genetic analysis and similarity to the genetic profile previously described by Portrat-Doyen S. and cols. together with the patient's clinical presentation strongly supported the diagnosis of AS deficiency type I for the first time in a Portuguese patient (25).

## CONCLUSIONS

AS deficiency is a rare case of hyperreninemic hypoaldosteronism inherited in an autosomal recessive pattern. This condition represents a disorder of terminal biosynthesis of aldosterone with a typical manifestation of salt-wasting disorder in early life. Proper diagnosis of AS deficiency is essential for correct lifelong management, and genetic confirmation of *CYP11B2* mutation allows for evaluation of familial adult members, information regarding the implications on offspring and reducing potential life-threatening risks associated with the disease.

This is the first report of a Portuguese patient with presumed AS deficiency type I in whom the molecular basis has been clarified, matching the clinical and laboratory diagnosis initially suspected.

Mineralocorticoid therapy allowed remarkable weight recovery and total resolution of the initial symptoms and laboratory abnormalities. Expectedly, during adulthood, these patients present a favourable clinical course despite no fludrocortisone therapy and may only show an asymptomatic persistent abnormal steroid pattern throughout life.

## References

[1] Hui E, Yeung MC, Cheung PT, Kwan E, Low L, Tan KC, et al. The clinical significance of aldosterone synthase deficiency: report of a novel mutation in the CYP11B2 gene. BMC endocrine disorders. 2014;14(1):29. 10.1186/1472-6823-14-29 PMC3976226 24694176

[2] Root AW. Disorders of aldosterone synthesis, secretion, and cellular function. Curr Opin Pediatr. 2014;26(4):480-6. 10.1097/MOP.0000000000000104 24840884

[3] White PC. Aldosterone synthase deficiency and related disorders. Mol Cell Endocrinol. 2004;217(1):81-7. 10.1016/j.mce.2003.10.013 15134805

[4] Collinet E, Pelissier P, Richard O, Gay C, Pugeat M, Morel Y, et al. [Four cases of aldosterone synthase deficiency in childhood]. Arch Pediatr. 2012;19(11):1191-5. 10.1016/j.arcped.2012.08.018 23062999

[5] Katznelson D, Sack J, Kraiem Z, Lunenfeld B. Congenital hypoaldosteronism. Thirteen year follow-up in identical twins. Horm Res. 1979;11(1):22-8. 10.1159/000179034 225254

[6] Kayes-Wandover KM, Tannin GM, Shulman D, Peled D, Jones KL, Karaviti L, et al. Congenital hyperreninemic hypoaldosteronism unlinked to the aldosterone synthase (CYP11B2) gene. J Clin Endocrinol Metabol. 2001;86(11):5379-82. 10.1210/jcem.86.11.8005 11701710

[7] Kondo E, Nakamura A, Homma K, Hasegawa T, Yamaguchi T, Narugami M, et al. Two novel mutations of the CYP11B2 gene in a Japanese patient with aldosterone deficiency type 1. Endocr J. 2013;60(1):51-5. 10.1507/endocrj.ej12-0248 23018980

[8] Kuribayashi I, Kuge H, Santa RJ, Mutlaq AZ, Yamasaki N, Furuno T, et al. A missense mutation (GGC [435Gly]→ AGC [Ser]) in exon 8 of the CYP11B2 gene inherited in Japanese patients with congenital hypoaldosteronism. Hormone Res. 2003;60(5):255-60. 10.1159/000074041 14614232

[9] Leshinsky-Silver E, Landau Z, Unlubay S, Bistrizer T, Zung A, Tenenbaum-Rakover Y, et al. Congenital hyperreninemic hypoaldosteronism in Israel: sequence analysis of CYP11B2 gene. Hormone Res. 2006;66(2):73-8. 10.1159/000093583 16733366

[10] Peter M, Sippell WG. Congenital hypoaldosteronism: the Visser-Cost syndrome revisited. Pediatr Res. 1996;39(3):554-60. 10.1203/00006450-199603000-00027 8929880

[11] Peter M, Nikischin W, Heinz-Erian P, Fussenegger W, Kapelari K, Sippell W. Homozygous deletion of arginine-173 in the CYP11B2 gene in a girl with congenital hypoaldosteronism. Corticosterone methyloxidase deficiency type II. Horm Res. 1998;50(4):222-5. 10.1159/000023278 9838244

[12] Peter M, Bunger K, Drop S, Sippell WG. Molecular genetic study in two patients with congenital hypoaldosteronism (types I and II) in relation to previously published hormonal studies. Eur J Endocrinol. 1998;139(1):96-100. 10.1530/eje.0.1390096 9703385

[13] Rubio-Cabezas O, Regueras L, Muñoz-Calvo M, Bartolomé M, Pozo J, Argente J. Primary hypoaldosteronism and moderate bilateral deafness in a child with a homozygous missense mutation (Thr318Met) in the CYP11B2 gene. An Pediatr (Barc). 2010;73(1):31-4. 10.1016/j.anpedi.2010.04.008 20639134

[14] Üstyol A, Atabek ME, Taylor N, Yeung MC, Chan AO. Corticosterone methyl oxidase deficiency type 1 with normokalemia in an infant. J Clin Res Pediatr Endocrinol. 2016;8(3):356-9. 10.4274/jcrpe.2824 PMC5096503 27125267

[15] Wikiera B, GŁa̧b E, Barg E, Noczyńska A. Congenital hypoaldosteronism associated with nails hypothrophy – case report and review of the literature. Pediatr Endocrinol Diabetes Metab. 2008;14(4):253-6. 19239795

[16] Rösler A. The natural history of salt-wasting disorders of adrenal and renal origin. J Clin Endocrinol Metab. 1984;59(4):689-700. 10.1210/jcem-59-4-689 6384251

[17] Rösler A, Rabinowitz D, Theodor R, Ramirez LC, Ulick S. The nature of the defect in a salt-wasting disorder in Jews of Iran. J Clin Endocrinol Metab. 1977;44(2):279-91. 10.1210/jcem-44-2-279 838841

[18] Nomoto S, Massa G, Mitani F, Ishimura Y, Miyahara K, Toda K, et al. CMO I deficiency caused by a point mutation in exon 8 of the human CYP11B2 gene encoding steroid 18-hydroxylase (P450C18). Biochem Biophys Res Commun. 1997;234(2):382-5. 10.1006/bbrc.1997.6651 9177280

[19] Pascoe L, Curnow KM, Slutsker L, Rösler A, White PC. Mutations in the human CYP11B2 (aldosterone synthase) gene causing corticosterone methyloxidase II deficiency. Proc Natl Acad Sci U S A. 1992;89(11):4996-5000. 10.1073/pnas.89.11.4996 PMC49215 1594605

[20] Rainey WE. Adrenal zonation: clues from 11beta-hydroxylase and aldosterone synthase. Mol Cell Endocrinol. 1999;151(1-2):151-60. 10.1016/s0303-7207(99)00051-9 10411330

[21] Dtattani MT, Gevers EF. Endocrinology of fetal development. In: Shlomo Melmed KSP, Larsen PR, Kronenberg HM, editors. Williams textbook of endocrinology. 13th ed. Philadelphia: Elsevier; 2016.

[22] Freel EM, Ingram M, Wallace AM, White A, Fraser R, Davies E, et al. Effect of variation in CYP11B1 and CYP11B2 on corticosteroid phenotype and hypothalamic-pituitary-adrenal axis activity in hypertensive and normotensive subjects. Clin Endocrinol (Oxford). 2008;68(5):700-6. 10.1111/j.1365-2265.2007.03116.x 17980006

[23] Ulick S, Gautier E, Vetter KK, Markello JR, Yaffe S, Lowe CU. An aldosterone biosynthetic defect in a salt-losing disorder. J Clin Endocrinol Metab. 1964;24:669-72. 10.1210/jcem-24-7-669 14212087

[24] Baranowski ES, Arlt W, Idkowiak J. Monogenic disorders of adrenal steroidogenesis. Hormone Res. 2018;89(5):292-310. 10.1159/000488034 PMC6067656 29874650

[25] Portrat-Doyen S, Tourniaire J, Richard O, Mulatero P, Aupetit-Faisant B, Curnow KM, et al. Isolated aldosterone synthase deficiency caused by simultaneous E198D and V386A mutations in the CYP11B2 gene. J Clin Endocrinol Metab. 1998;83(11): 4156-61. 10.1210/jcem.83.11.5258 9814506

